# Pneumonia scoring systems for severe COVID-19: which one is better

**DOI:** 10.1186/s12985-021-01502-6

**Published:** 2021-02-10

**Authors:** PengFei Cheng, Hao Wu, JunZhe Yang, XiaoYang Song, MengDa Xu, BiXi Li, JunJun Zhang, MingZhe Qin, Cheng Zhou, Xiang Zhou

**Affiliations:** 1 Department of Anesthesiology, General Hospital of Central Theater Command of PLA, Wuhan, China; 2 Department of Gastroenterology, General Hospital of Central Theater Command of PLA, Wuhan, China; 3 Department of Radiation Oncology, Nanfang Hospital, Southern Medical University, Guangzhou, China

**Keywords:** Novel coronavirus pneumonia, Disease assessment, APACHE II score, MuLBSTA score, CURB-65 score

## Abstract

**Purpose:**

To investigate the predictive significance of different pneumonia scoring systems in clinical severity and mortality risk of patients with severe novel coronavirus pneumonia.

**Materials and methods:**

A total of 53 cases of severe novel coronavirus pneumonia were confirmed. The APACHE II, MuLBSTA and CURB-65 scores of different treatment methods were calculated, and the predictive power of each score on clinical respiratory support treatment and mortality risk was compared.

**Results:**

The APACHE II score showed the largest area under ROC curve in both noninvasive and invasive respiratory support treatment assessments, which is significantly different from that of CURB-65. Further, the MuLBSTA score had the largest area under ROC curve in terms of death risk assessment, which is also significantly different from that of CURB-65; however, no difference was noted with the APACHE II score.

**Conclusion:**

For patients with COVID, the APACHE II score is an effective predictor of the disease severity and mortality risk. Further, the MuLBSTA score is a good predictor only in terms of mortality risk.

## Introduction

A novel coronavirus pneumonia outbreak in Wuhan, China, in December 2019, has had a major impact globally. This disease was named as "Corona Virus Disease 2019" (COVID-19), and this new type of corona virus was named as SARS-CoV-2 by the World Health Organization. According to the 7th edition of the Chinese National Health Commission, such patients can be categorized into light, normal, and severe depending on their clinical symptoms and test results [1]. However, a proper methodology to reflect the degree of the disease and predict the disease development still does not exist.

The CURB-65 (confusion, urea, respiratory rate, blood pressure, and age 65) scoring system [2] is being used as a measure of the severity of community-acquired pneumonia, which can be combined with other clinical parameters to assess if the patients need to be hospitalized or transferred to the intensive care unit (ICU). The APACHE II (acute physiology and chronic health evaluation II) scoring system [3, 4] is being used to evaluate the condition of patients in ICU using 12 parameters. Currently, this method is being widely used clinically due to its capability of distinguishing the severity of the disease. The MuLBSTA (multilobular infiltration, hypo-lymphocytosis, bacterial coinfection, smoking history, hyper-tension, and age) scoring system is an easy-to-use clinical tool to predict the risk of mortality in high-risk and low-risk groups of patients with viral pneumonia. With the use of this method, hospitalized patients with viral pneumonia can be classified into relevant risk categories to acquire guidance for further clinical decision making [5]. However, the effectiveness of these three scoring systems in assessing COVID-19 has not yet been reported.

## Methods

### Inclusion criteria

**Fig. 1:**
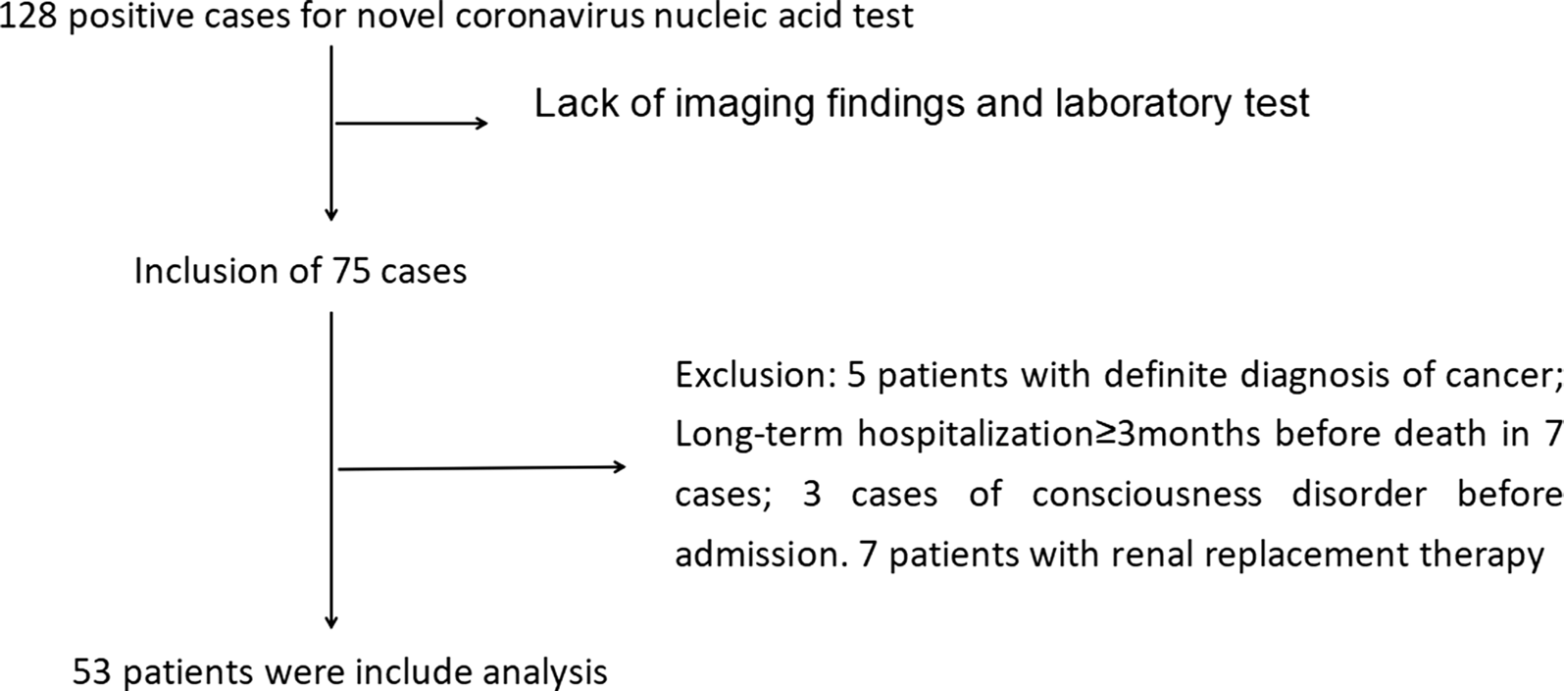
Research process

This single-center, retrospective observational clinical study was approved by the Ethics Committee of the General Hospital of central theater command of PLA (2020–008-1). A total of 53 cases of severe novel coronavirus pneumonia were confirmed in the General Hospital of central theater command of People’s Liberation Army between 1, January 2020 and 4, March 2020 (Fig. 1).

The inclusion criteria for the study were as follows: (1) All patients were confirmed positively by SARS-CoV-2 nucleic acid RT-PCR (Ct value ≤ 38.0, BGI, Shenzhen, China) using specimens derived from oropharyngeal swabs or sputum, prior to or during the hospitalization; and (2) Patients with the severe form of the disease were categorized based on the 7th edition of the Chinese National Health Commission, which included meeting any of the following criteria: (1) shortness of breath, respiratory rate ≥ 30 beats/min; (2) oxygen saturation ≤ 93% in the resting state; (3) arterial blood oxygen partial pressure (PaO_2_)/oxygen concentration (FiO_2_) ≤ 300 mmHg (1 mmHg = 0.133 kPa); and (4) lung images showing obvious progress of lesions > 50% within 24–48 h.

The exclusion criteria for the study were as follows: (1) Age < 18 years; (2) Patients with definite diagnosis of cancer; (3) Long-term hospitalization ≥ 3 m before death; (4) Presence of unconsciousness before admission; and (5) Patients receiving renal replacement therapy.

### Data acquisition

Data related to demography, underlying comorbidities, symptoms, physical and radiological findings, laboratory values, and respiratory and physiologic parameters of the subjects while receiving mechanical ventilation were collected from electronic and paper medical records. We used a positive bacterial culture of blood and sputum samples as the criteria for bacterial growth. The APACHE II, MuLBSTA, and CURB-65 scores were calculated for different treatment time points, and the predictive power of each score for treatment with clinical respiratory support and respective mortality risk was compared.

### Observational indicators

High-flow oxygen inhalation, noninvasive ventilator support, and invasive ventilator support were used as the three treatment methods. The APACHE II, MuLBSTA, and CURB-65 scoring systems were used to calculate the patient scores at each time point. The area under the receiver operating characteristic (ROC) curve was used to calculate the hierarchical boundary values of each scoring model for each treatment method [6]. The sensitivity and specificity of all the values were calculated, and the difference in area under ROC curve (AUROC) of each scoring model for the same treatment was compared. The patients were divided into high-flow oxygen inhalation group, noninvasive ventilator support group, and invasive ventilator support group. They were also categorized into death and non-death groups. The categorization was based on the severity of the patient's condition and the outcome. The APACHE II, MuLBSTA, and CURB-65 scores for the high-flow oxygen inhalation, noninvasive ventilator support, and invasive ventilation support groups prior to intubation were recorded. Further, the APACHE II, MuLBSTA, and CURB-65 scores in the death group were recorded on the day of death.

The MuLBSTA score was recorded based on the following [5]: multilobular infiltration (5 points), lymphocytes ≤ 0.8*109/L (4 points), bacterial infection (4 points), acute smokers (3 points) or quitters (2 points), hypertension (2 points), age ≥ 60 years (2 points), maximum 22 points.The CURB-65 score was recorded based on the following[2]: consciousness disorder (1 point), blood urea nitrogen > 7 mmol/L (1 point), respiratory frequency ≥ 30 beats/min (1 point), systolic blood pressure < 90 mmHg or diastolic blood pressure ≤ 60 mmHg (1 point), age ≥ 65 years (1 point), maximum 4 points. APACHE II score system is now widely used in the intensive care unit (ICU). APACHE II score system includes a 12-point acute physiology score (including Temperature, Heart rate, Breathing rate, Blood pressure, Oxygen partial pressure, PH, K^+^, Na^+^, Creatinine, HCT, WBC and Consciousness), Age point, and Chronic health evaluation. The higher the score, the more serious the condition [3, 4].

### Statistical methods

Software Package for Social Sciences (IBM SPSS 25.0) was used for statistical analysis. Dates were described with median and range of continuous variables as well as frequency and percentage of categorical variables. The performance of each scoring system was evaluated by measuring the AUROC. Further, the χ^2^ test was used to calculate sensitivity and specificity. The different scoring models used different ROC curve areas for comparison.

## Results

### Basic information

Out of the 53 patients, 27 patients in the high-flow nasal catheter oxygen therapy group were cured and discharged. The remaining 26 patients underwent noninvasive ventilator support. Out of these, 20 patients further underwent endotracheal intubation; however, 16 patients could not be cured and eventually died. One of the patients who died had only received noninvasive ventilator treatment but not endotracheal intubation. The median time from onset to admission was 7 days, onset to noninvasive ventilator support was 12 days, onset to invasive ventilator support was 20 days, onset to death was 25 days, and onset to discharge was 35 days. The other demographic characteristics are listed in Table 1.

**Table 1:** Demographic characteristics

	Patients
Age	61 (20–96)
Gender	
Male	36 (67.9%)
Female	17 (33.1%)
BMI	25.8 (19.6–30.5)
Chronic diseases	23 (43.4%)
Hypertension	20 (37.7)
Diabetes	9 (17)
Heart disease	11 (20.8)
Cerebrovascular disease	3 (5.7)
Treatment and outcome	
Noninvasive ventilator support	26 (49.1%)
Invasive ventilator support	20 (37.7%)
Cured	37 (69.8%)
Death	16 (30.2%)
Course of disease	
Onset to admission	7 (1–31)
Onset to noninvasive ventilator support	12 (6–32)
Onset to invasive ventilator support	20 (9–38)
Onset to death	25 (10–44)
Onset to discharge	35 (7–53)

### The scores of CURB-65, APACHE II, MuLBSTA, and the frequency of each score in each group

**Fig. 2:**
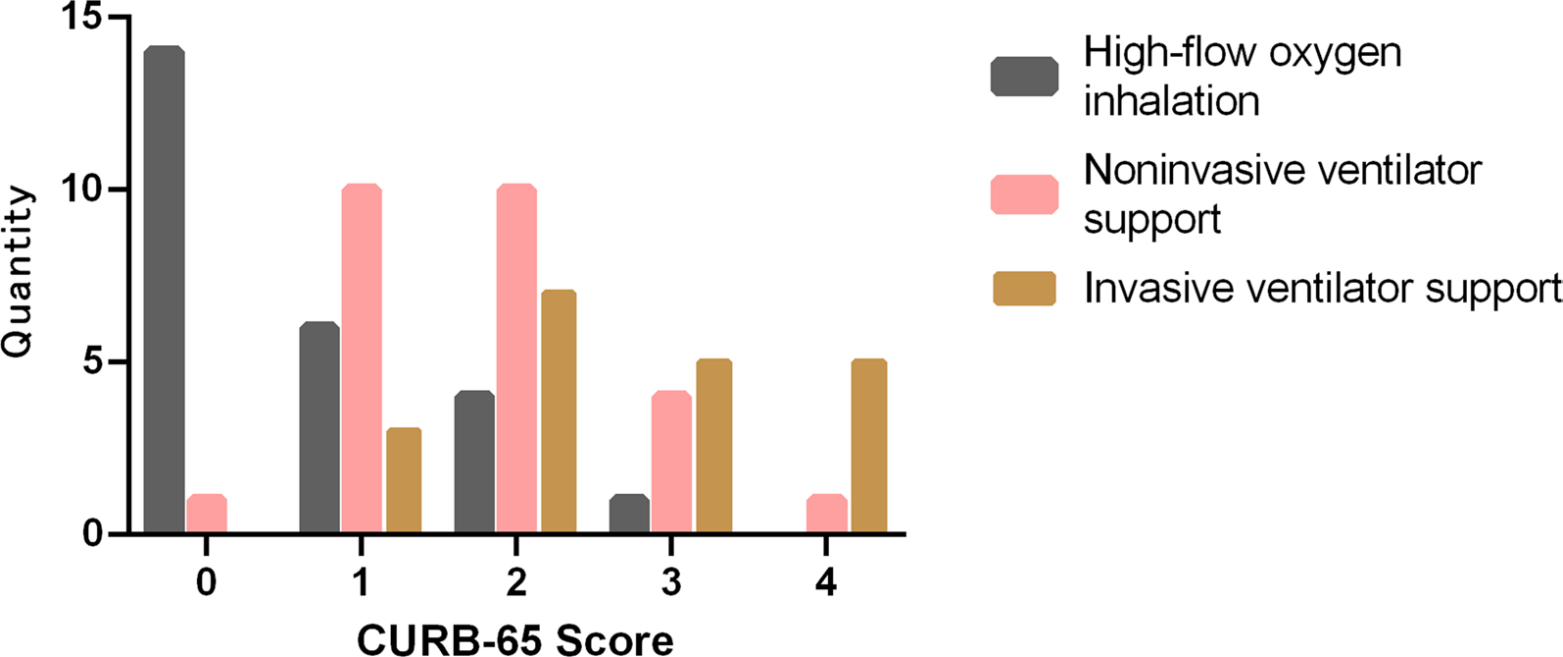
Distribution of different treatment methods in CURB-65 scoring system. Quantity means “number of patients”

**Fig. 3:**
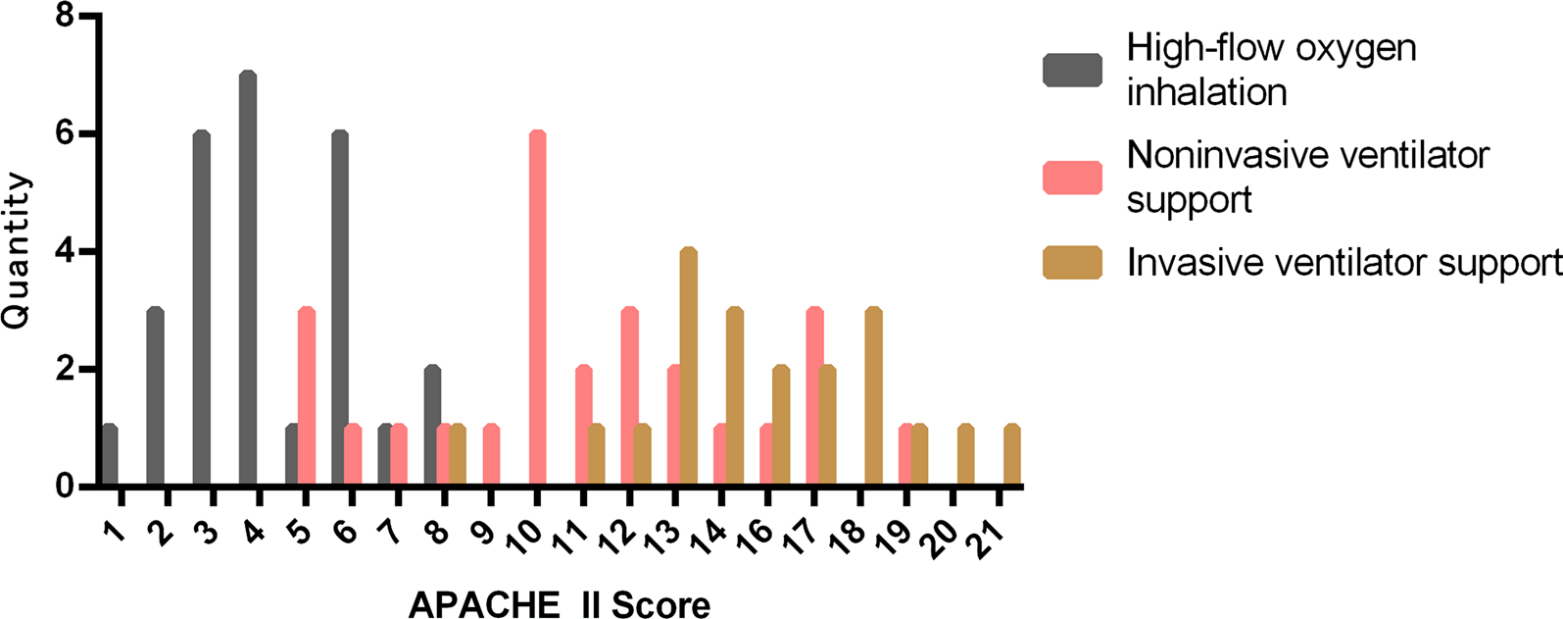
Distribution of different treatment methods in APACHE II scoring system. Quantity means “number of patients”

**Fig. 4:**
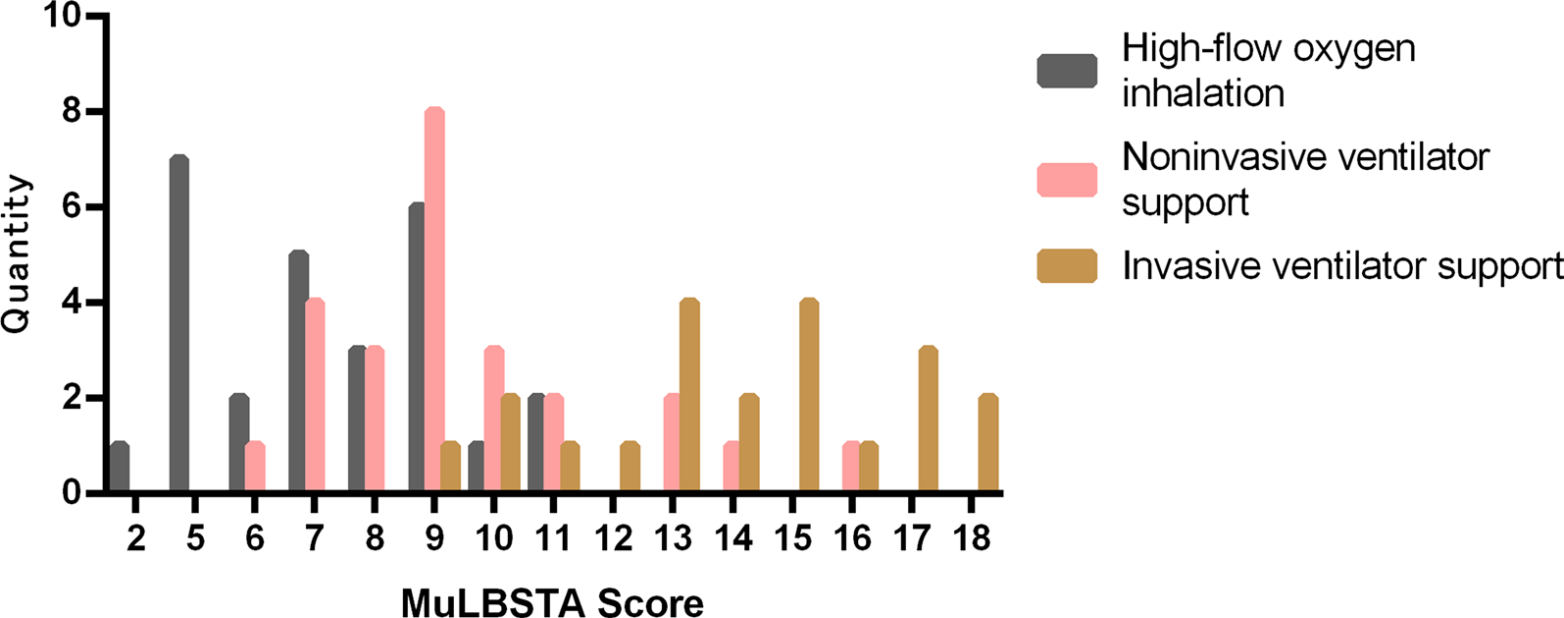
Distribution of different treatment methods in MuLBSTA scoring system. Quantity means “number of patients”

The frequency (number of patients) of high-flow oxygen inhalation group, noninvasive ventilation support group, and invasive ventilation support group in CURB score 0, 1, 2, 3, 4, in APACHE II score 1, 2, 3, 4, 5, 6, 7, 8, 9, 10, 11, 12, 13, 14, 16, 17, 19, 20, 21 and in MuLBSTA score 2, 5, 6, 7, 8, 9, 10, 11, 12, 13, 14, 15, 16, 17, 18 are separately shown in Figs. 2, 3, and 4.

### Cut-off values of CURB-65, APACHE II, and MuLBSTA for predicting the risk of noninvasive ventilator support, invasive ventilator support, and mortality

In terms of the cut-off values of CURB-65, 1.5 points was used for noninvasive ventilator support, 2.5 points for invasive ventilator support and mortality. In terms of the cut-off values of APACHE II, 9.5 points was used for noninvasive ventilator support, 12.5 points for invasive ventilator support and 11.5 points for mortality. In terms of the cut-off values of MuLBSTA, 8.5 points was used for noninvasive ventilator support, 10.5 points for invasive ventilator support and 13.5 points for mortality. These have been listed in Table 2.

**Table 2:** Cut-off values of CURB-65, APACHE II, and MuLBSTA for predicting the risk of noninvasive ventilator support, invasive ventilator support, and mortality

	Cut-off value	Youden index	ROC Area	Sensitivity	Specificity
Noninvasive ventilator support					
CURB-65 score	1.5	48.00	0.7493	0.6818	0.6452
APACHE II score	9.5	76.92	0.9459	1.0000	0.7941
MuLBSTA score	8.5	32.62	0.7560	0.6667	0.6923
Invasive ventilator support					
CURB-65 score	2.5	54.69	0.8561	0.5556	0.7143
APACHE II score	12.5	83.93	0.9297	0.9286	0.9231
MuLBSTA score	10.5	73.78	0.9235	0.6800	0.8929
Death					
CURB-65 score	2.5	46.11	0.7829	0.5833	0.7805
APACHE II score	11.5	73.83	0.9046	0.6190	0.9063
MuLBSTA score	13.5	94.59	0.9856	0.8462	0.8750

### Comparison of area under ROC curve of three scoring models in each group

**Fig. 5:**
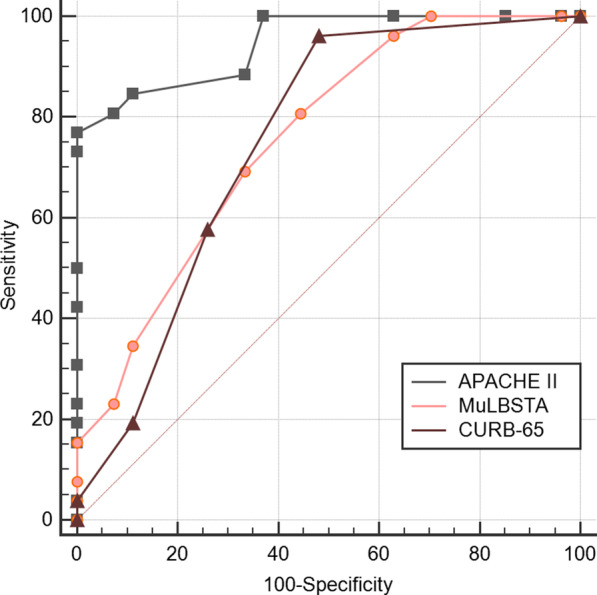
Comparison of noninvasive support ventilation with different scoring methods. The AUROC of the APACHE II scoring model was identified to be the largest and statistically different from that of the MuLBSTA and CURB-65 models (*P* = 0.0046 and 0.0059, respectively)

**Fig. 6:**
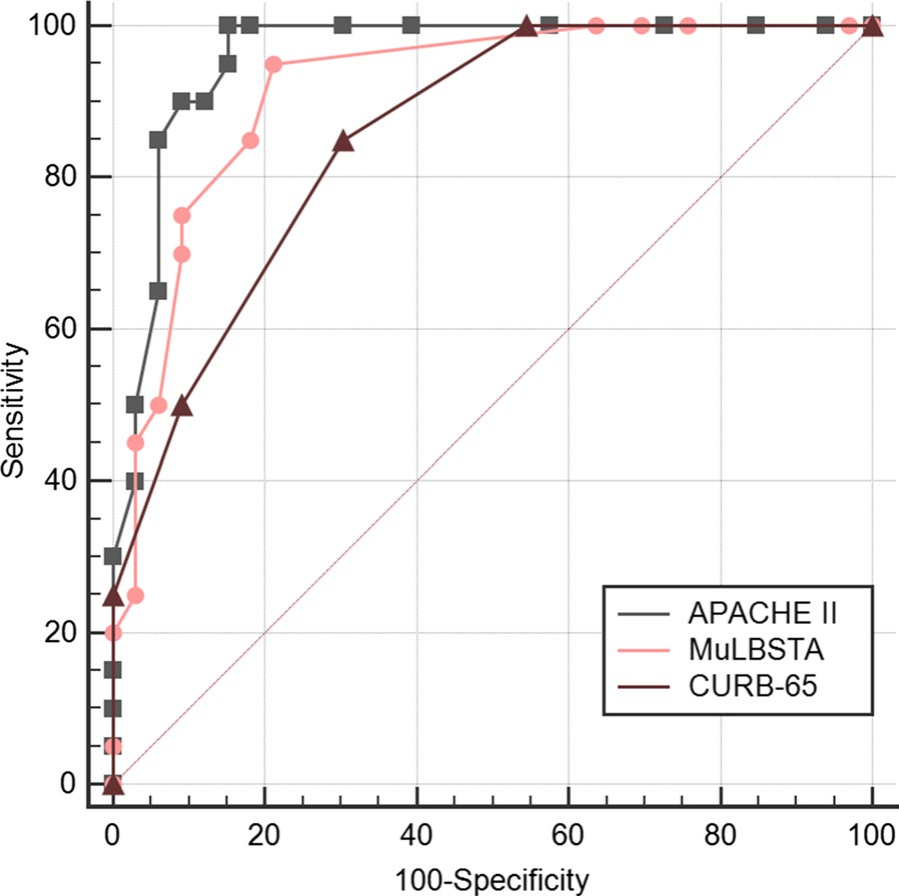
Comparison of invasive support ventilation with different scoring methods. The AUROC of the APACHE II scoring model was the largest, statistically different from the CURB-65 scoring model (*P* = 0.0372), and identical with the MuLBSTA scoring model (*P* = 0.2708)

**Fig. 7:**
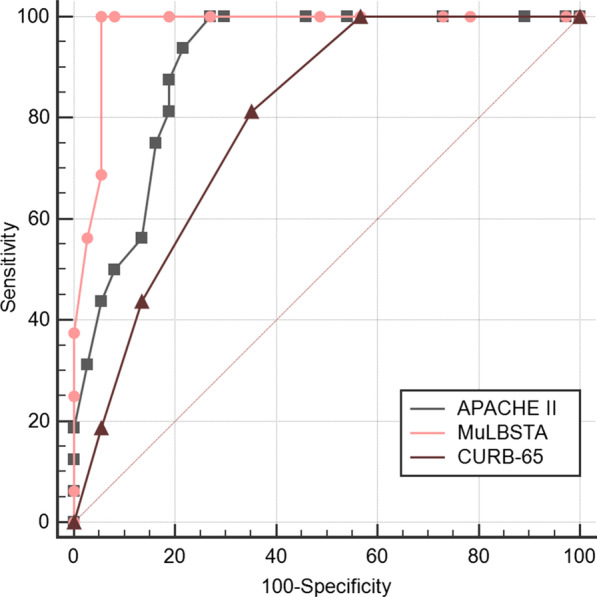
Comparison of mortality risk with different scoring methods. The AUROC of the MuLBSTA scoring model was identified to be the largest, which was statistically different from CURB-65 (*P* = 0.0021), and no difference was noted with APACHE II (P = 0.0549)

On evaluating the three scoring models for noninvasive ventilator support, the area under the ROC curve of the APACHE II scoring model was identified to be the largest and statistically different from that of the MuLBSTA and CURB-65 models (*P* = 0.0046 and 0.0059, respectively). Further, no statistical difference was identified between the MuLBSTA and CURB-65 models (*P* = 0.9369). The assessment of the need for invasive ventilator support revealed that the AUROC of the APACHE II scoring model was the largest, statistically different from the CURB-65 scoring model (*P* = 0.0372), and identical with the MuLBSTA scoring model (*P* = 0.2708). When assessing mortality, the AUROC of the MuLBSTA scoring model was identified to be the largest, which was statistically different from CURB-65 (*P* = 0.0021). However, no difference was noted with APACHE II (*P* = 0.0549). These findings are listed in Table 3 and shown in Figs. 5, 6, and 7.

**Table 3:** Comparison of the area under the ROC curve between the three scoring models

	Z value	*P* value
Noninvasive ventilator support		
APACHE II vs. MuLBSTA	2.837	0.0046
APACHE II vs. CURB-65	2.754	0.0059
MuLBSTA vs. CURB-65	0.0791	0.9369
Invasive ventilator support		
APACHE II vs. MuLBSTA	1.101	0.2708
APACHE II vs. CURB-65	2.084	0.0372
MuLBSTA vs. CURB-65	1.054	0.2921
Death		
APACHE II vs. MuLBSTA	1.920	0.0549
APACHE II vs. CURB-65	2.433	0.0150
MuLBSTA vs. CURB-65	3.072	0.0021

### Multivariate analysis of individual risk factors in each model for DEATH and INTUBATION in patients with COVID-19

On evaluating the individual risk factors in each model for death in patients with COVID-19, bacterial coinfection and age ≥ 60 years from MuLBSTA scoring model, breathing rate ≥ 30/min and age ≥ 65 years from CURB-65 scoring model were considered to be statistically different (*P* < 0.05); On evaluating the individual risk factors in each model for intubation in patients with COVID-19, breathing rate ≥ 30/min from CURB-65 scoring model were considered to be statistically different (*P* < 0.05). All other individual risk factors from the three scoring models had no statistical differences. These have been listed in Tables 4 and 5.

**Table 4:** Multivariate analysis of individual risk factors in each model for DEATH in patients with COVID-19

Risk factors	*P* value	OR value
MuLBSTA Score (sensitivity 0.8462, specificity 0.8750)		
Multilobular infiltrates	1.000	–
Lymphocyte ≤ 0.8 * 10^ 9^/L	0.211	–
Bacterial coinfection	0.010	12.457
Quit-smoker or acute-smoker	0.276	–
Hypertension	0.565	–
Age ≥ 60 years	0.014	12.220
CURB-65 Score (sensitivity 0.5833, specificity 0.7805)		
Disturbance of consciousness	0.522	–
Blood urea nitrogen > 7 mmol/L	0.379	–
Breathing rate ≥ 30/min	0.018	9.351
Systolic pressure < 90 mmHg or diastolic pressure ≤ 60 mmHg	1.000	–
Age ≥ 65 years	0.016	11.591
APACHEII Score (severity 0.9286, specificity 0.9231)		
Temperature	0.942	–
Heart rate	0.996	–
Breathing rate	0.158	–
Blood pressure	1.000	–
Oxygen partial pressure	0.996	–
PH	0.487	–
K^+^	0.273	–
Na^+^	0.174	–
Creatinine	0.352	–
HCT	0.641	–
WBC	0.382	–
Consciousness	0.596	–
Age	0.995	–
Chronic diseases	0.586	–

**Table 5:** Multivariate analysis of individual risk factors in each model for INTUBATION in patients with COVID-19

Risk factors	*P* value	OR value
MuLBSTA Score (sensitivity 0.6800, specificity 0.8929)		
Multilobular infiltrates	1.000	–
Lymphocyte ≤ 0.8 * 10^ 9^/L	0.998	–
Bacterial coinfection	0.997	–
Quit-smoker or acute-smoker	0.419	–
Hypertension	0.118	–
Age ≥ 60 years	0.247	–
CURB-65 Score (sensitivity 0.5556, specificity 0.7143)		
Disturbance of consciousness	0.055	–
Blood urea nitrogen > 7 mmol/L	0.241	–
Breathing rate ≥ 30/min	0.025	7.48
Systolic pressure < 90 mmHg or diastolic pressure ≤ 60 mmHg	1.000	–
Age ≥ 65 years	0.144	–
APACHEII Score (sensitivity 0.6190, specificity 0.9063)		
Temperature	0.169	–
Heart rate	0.153	–
Breathing rate	0.051	–
Blood pressure	1.000	–
Oxygen partial pressure	0.219	–
PH	0.070	–
K^+^	0.895	–
Na^+^	0.168	–
Creatinine	0.692	–
HCT	0.503	–
WBC	0.498	–
Consciousness	0.987	–
Age	0.075	–
Chronic diseases	0.400	–

## Discussion

In this study, we analyzed 53 patients with a severe form of the disease in our hospital. These patients were tested positive for the nucleic acid test between January 2020 and February 2020. In terms of demographic characteristics, the patients were older, mostly male, and had underlying diseases similar to those described by other scholars [7]. However, Wang et al. identified that among the COVID patients, 54.3% were male and 45.7% were female, showing no gender difference [8]. Only severe cases were included in our study; the rate of patients on noninvasive ventilator support, patients on invasive ventilator support, and mortality was identified to be 49.1%, 37.7%, and 30%, respectively, which was similar to the results of other studies [9, 10]. The median time from onset to admission was 7 days, onset to noninvasive ventilator treatment was 12 days, and onset to invasive ventilator treatment was 20 days. The obtained data were found to be similar to that of previous studies [11, 12]. The median time from onset to discharge was 35 days and from onset to death was 25 days.

According to current studies, early respiratory support treatment can improve the condition of patients with severe COVID-19 [1]. However, such treatments are normally administered based on a single test or simple clinical experience of doctors, which has significant limitations. In our study, three scoring systems are used to calculate the approximate scores of each respiratory treatment method. This can help clinicians judge and perform reasonable and timely respiratory management. Our research suggests that high-flow oxygen inhalation can be considered when the APACHE II score < 9.5, MuLBSTA score < 8.5, or CURB-65 score < 1.5. Further, noninvasive ventilator support can be considered when the APACHE II score ranges from 9.5 to 12.5, MuLBSTA score ranges from 8.5 to 10.5, or CURB-65 score ranges from 1.5 to 2.5, and invasive ventilator support can be considered when the APACHE II score > 12.5, MuLBSTA score ≥ 10.5, or CURB-65 score ≥ 2.5. Patients may be at risk of death when the APACHE II score > 11.5, MuLBSTA score > 13.5, or CURB-65 score > 2.5.

The APACHE II score is a classic tool for assessing the severity of the disease in patients in the ICU [4, 13]. The higher the score, the more critical the situation, worse the prognosis, and higher the mortality [13, 14]. Wang et al. determined that the median APACHE II score of patients with severe novel coronavirus pneumonia was 17 (10–22) [8], which is also consistent with our research. The APACHE II score is better than the scores of the other two methods when evaluating noninvasive respiratory support treatment (*P* = 0.0046 and 0.0059, respectively). In terms of invasive respiratory support therapy, the APACHE II score is better than the CURB-65 score (*P* = 0.0372). Further, the APACHE II score is also better than that of CURB-65 (*P* = 0.0150) in predicting mortality risk. Therefore, with comprehensive consideration, the APACHE II score is first recommended when assessing the overall condition of patients with COVID.

The MuLBSTA score assesses the risk of death from viral pneumonia [5, 15]. Patients with MuLBSTA score > 12 are categorized as the high-risk group [7]. Further, in our study, the patients are at risk of death when MuLBSTA score > 13.5. The MuLBSTA score has a sensitivity of 0.6364 and specificity of 0.9355 when assessing the risk of death, as reported through studies [5]. We also identify the MuLBSTA score to be better compared to the CURB-65 score in assessing death risk (*P* = 0.0021). Therefore, we recommend MuLBSTA score as the first choice when predicting only the risk of death.

The CURB-65 score is often used to assess the severity of community-acquired pneumonia, which requires only few assessment tools [16]. Owing to its simplicity and low score, the CURB-65 score has high sensitivity and low specificity when assessing a condition [17]. It is necessary to combine other parameters of the patient with the CURB-65 score to reach a final clinical judgment. Similarly, in our study, we find that the CURB-65 score is not as efficient as the other two scoring systems in assessing the necessity of respiratory support and death risk. Therefore, based on our study results, the CURB-65 score is not recommended for assessment of patients with COVID.

Our study has few limitations. It is a single-center, retrospective study with a relatively small sample size. Further, there is a certain degree of clinical data deficiency.

## Conclusion

The current study find that for patients with COVID, the APACHE II score is an effective predictor of the disease severity and mortality risk, whereas, the MuLBSTA score is only a good predictor of mortality risk.

## Abbreviations

APACHE II: Acute physiology and chronic health evaluation II
AUROC: Area under ROC curve
SPSS: Software Package for Social Sciences
2019-nCoV: Novel corona virus
COVID-19: Corona Virus Disease 2019
CURB-65: Confusion, urea, respiratory rate, blood pressure, and age 65
ICU: Intensive care unit
MuLBSTA: Multilobular infiltration, hypo-lymphocytosis, bacterial coinfection, smoking history, hyper-tension, and age
ROC: Receiver operating characteristic

## Availability of data and material

The datasets used or analysed during the current study are available from the corresponding author on reasonable request.

## References

[1] National Health and Health Commission of the People's Republic of China. Diagnosis and Treatment of Pneumonia of New Coronavirus Infection (Trial Version 7). (2020-03-03). http://www.nhc.gov.cn/yzygj/s7653p/202003/46c9294a7dfe4cef80dc7f5912eb1989.shtml.

[2] Barlow G, Nathwani D, Davey P (2007). The CURB65 pneumonia severity score outperforms generic sepsis and early warning scores in predicting mortality in community-acquired pneumonia. Thorax.

[3] Ferrer M, Travierso C, Cilloniz C, Gabarrus A, Ranzani OT, Polverino E, Liapikou A, Blasi F, Torres A (2018). Severe community-acquired pneumonia: characteristics and prognostic factors in ventilated and non-ventilated patients. PLoS ONE.

[4] Godinjak A, Iglica A, Rama A, Tančica I, Jusufović S, Ajanović A, Kukuljac A (2016). Predictive value of SAPS II and APACHE II scoring systems for patient outcome in a medical intensive care unit. Acta Med Acad.

[5] Guo L, Wei D, Zhang X, Wu Y, Li Q, Zhou M, Qu J (2019). Clinical features predicting mortality risk in patients with viral pneumonia: the MuLBSTA Score. Front Microbiol.

[6] Ruan L, Chen GY, Liu Z, Zhao Y, Xu GY, Li SF, Li CN, Chen LS, Tao Z (2018). The combination of procalcitonin and C-reactive protein or presepsin alone improves the accuracy of diagnosis of neonatal sepsis: a meta-analysis and systematic review. Crit Care (Lond, Engl).

[7] Chen N, Zhou M, Dong X, Qu J, Gong F, Han Y, Qiu Y, Wang J, Liu Y, Wei Y, Xia J, Yu T, Zhang X, Zhang L (2020). Epidemiological and clinical characteristics of 99 cases of 2019 novel coronavirus pneumonia in Wuhan, China: a descriptive study. Lancet (London, England).

[8] Wang D, Hu B, Hu C, Zhu F, Liu X, Zhang J, Wang B, Xiang H, Cheng Z, Xiong Y, Zhao Y, Li Y, Wang X, Peng Z (2020). Clinical characteristics of 138 hospitalized patients with 2019 novel coronavirus-infected pneumonia in Wuhan, China. JAMA.

[9] Wang Y, Wang Y, Chen Y, Qin Q (2020). Unique epidemiological and clinical features of the emerging 2019 novel coronavirus pneumonia (COVID-19) implicate special control measures. J Med Virol.

[10] Mizumoto K, Chowell G (2020). Estimating risk for death from 2019 novel coronavirus disease, China, January–February 2020. Emerg Infect Dis.

[11] Huang C, Wang Y, Li X, Ren L, Zhao J, Hu Y, Zhang L, Fan G, Xu J, Gu X, Cheng Z, Yu T, Xia J, Wei Y, Wu W, Xie X, Yin W, Li H, Liu M, Xiao Y (2020). Clinical features of patients infected with 2019 novel coronavirus in Wuhan, China. Lancet (London, England).

[12] Guan WJ, Ni ZY, Hu Y, Liang WH, Ou CQ, He JX, Liu L, Shan H, Lei CL, Hui D, Du B, Li LJ, Zeng G, Yuen KY, Chen RC, Tang CL, Wang T, Chen PY, Xiang J, Li SY (2020). Clinical characteristics of coronavirus disease 2019 in China. N Engl J Med.

[13] Zhou XY, Ben SQ, Chen HL, Ni SS (2015). A comparison of APACHE II and CPIS scores for the prediction of 30-day mortality in patients with ventilator-associated pneumonia. Int J Infect Dis.

[14] Chen L, Han X, Li Y, Zhang C, Xing X (2020). Impact of early neuraminidase inhibitor treatment on clinical outcomes in patients with influenza B-related pneumonia: a multicenter cohort study. Eur J Clin Microbiol Infect Dis.

[15] Kalil AC, Thomas PG (2019). Influenza virus-related critical illness: pathophysiology and epidemiology. Crit Care (Lond, Engl).

[16] Brabrand M, Henriksen DP (2018). CURB-65 score is equal to NEWS for identifying mortality risk of pneumonia patients: an observational study. Lung.

[17] Murillo-Zamora E, Medina-González A, Zamora-Pérez L, Vázquez-Yáñez A, Guzmán-Esquivel J, Trujillo-Hernández B (2018). Performance of the PSI and CURB-65 scoring systems in predicting 30-day mortality in healthcare-associated pneumonia. Med Clin.

